# Revisiting the vulnerability of juvenile bigeye (*Thunnus obesus*) and yellowfin (*T*. *albacares*) tuna caught by purse-seine fisheries while associating with surface waters and floating objects

**DOI:** 10.1371/journal.pone.0179045

**Published:** 2017-06-29

**Authors:** Joe Scutt Phillips, Graham M. Pilling, Bruno Leroy, Karen Evans, Thomas Usu, Chi Hin Lam, Kurt M. Schaefer, Simon Nicol

**Affiliations:** 1 Climate Change Research Centre, University of New South Wales, Sydney, Australia; 2 Oceanic Fisheries Programme, The Pacific Community (SPC), Nouméa, New Caledonia; 3 The Commonwealth Scientific and Industrial Research Organisation Marine and Atmosphere Research, Hobart, Australia; 4 National Fisheries Authority, Port Moresby, Papua, New Guinea; 5 Large Pelagics Research Centre, University of Massachusetts, Gloucester, United States of America; 6 The Inter-American Tropical Tuna Commission, La Jolla, California, United States of America; 7 Australian Bureau of Agricultural and Resource Economics and Sciences, Canberra, Australia; University of Waikato, NEW ZEALAND

## Abstract

Tuna fisheries catch over three million tonnes of skipjack tuna (*Katsuwonus pelamis*) each year, the majority of which come from purse-seine vessels targeting fish associated with man-made fish aggregating devices (FADs). A significant challenge for fisheries management is to maximize the efficiency of skipjack tuna catches whilst minimizing the bycatch of small and immature bigeye (*Thunnus obesus*) and yellowfin (*T*. *albacares*) tuna, for which long-term sustainability is uncertain in 75% of the world’s stocks. To better manage the issues common with this fishing method, an improved understanding of tuna behaviour around FADs is necessary. We probabilistically classified the vertical behavioural patterns of 50 bigeye and 35 yellowfin tuna (mean fork length 72cm and 70cm, respectively) electronically tagged throughout the western and central Pacific Ocean into shallow and deep states, using a state-space modelling approach. The occurrence of surface-association behaviours, defined as an individual remaining in a shallow state for 24-hours, was examined in relation to known capture events and FAD density. In general, surface-association events for both species were short and lasted on average less than three days, although events as long as 28 days were observed, and were more common in yellowfin when in archipelagic waters. Events were longest immediately following tagging in 62% and 17% of bigeye and yellowfin, respectively. Surface-association behaviour was not generally estimated just prior to recapture, being either non-existent or shorter than two days for 85% of bigeye and 74% of yellowfin. Current management measures in purse-seine tuna fisheries involve periodic or spatial closures for FAD use. If the chief benefit to purse-seine fishers of surface-association around floating objects is in locating schools in horizontal space at short-term time-scales, rather than holding fish near the surface for extended periods, controlling the number of sets made on FADs should be explored further as an additional management tool.

## Introduction

**Fig 1:**
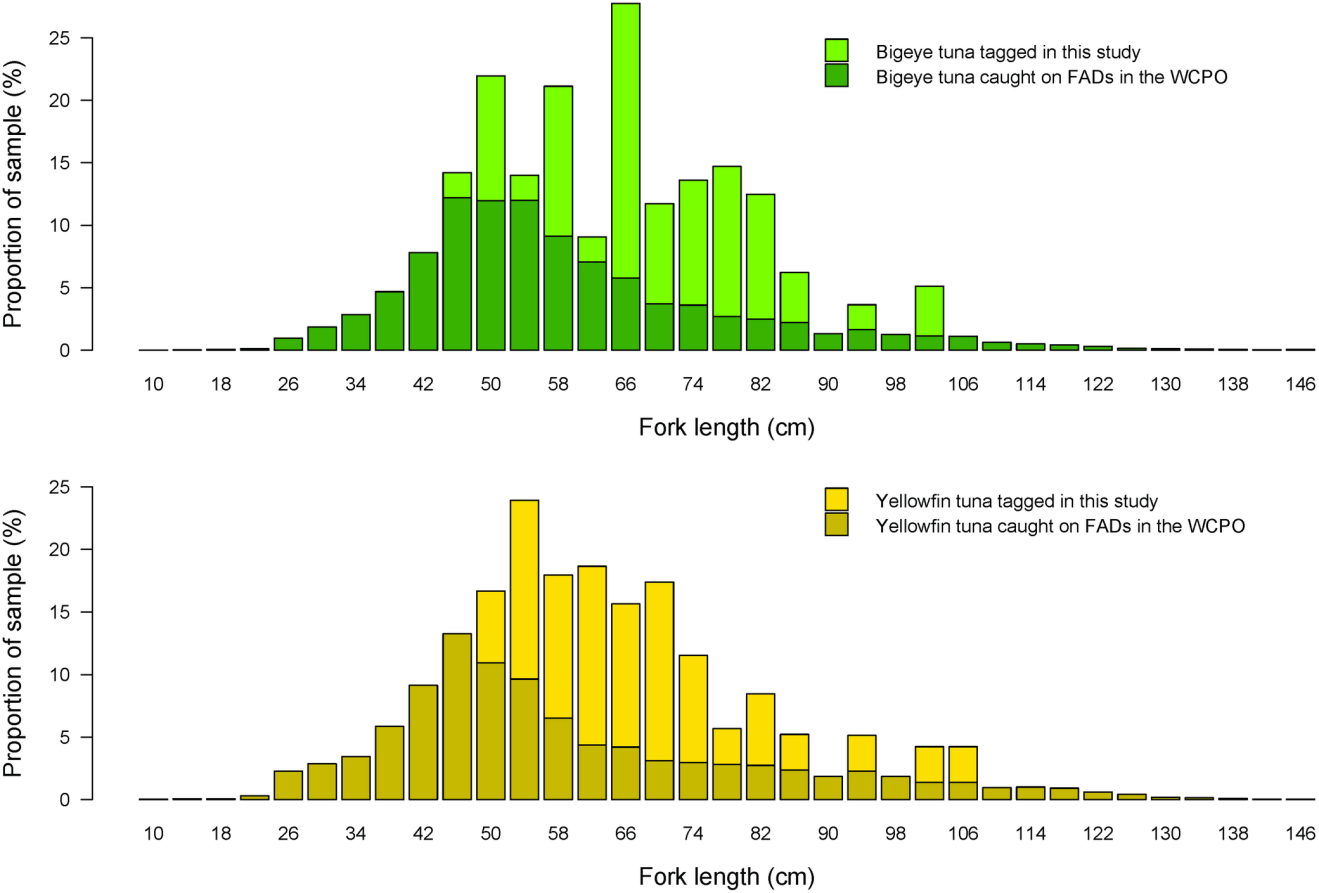
Size-classes of bigeye and yellowfin tuna. Proportion of all fork length size class samples by purse seine vessel observers made between 1994 and 2016, for bigeye (top) and yellowfin (bottom) tuna caught at anchored or drifting FADs in the WCPO. The same proportion of fork length size class at release is stacked above for our sample of electronically tagged fish from this study.

Industrial purse-seine fisheries for tuna are some of the largest globally [1], and purse-seine catches of tuna associated with floating objects in the Western and Central Pacific Ocean (WCPO) are the world’s highest [2]. The deployment of anchored and drifting man-made floating objects known as fish aggregation devices (FADs) to attract tropical tuna and improve catch efficiency has become common practice in the region and worldwide over the last 40 years [3–8]. Purse-seine fishers principally target skipjack tuna (*Katsuwonus pelamis*, Scombridae) schools. However, sets on floating objects often result in not only significant catches of this species, but also of small juvenile bigeye (*Thunnus obesus*, Scombridae) and yellowfin (*T*. *albacares*, Scombridae) tuna across an interquartile size range (IQR) of 49-69cm and 47-73cm fork length (FL), respectively (Fig 1), as aggregations of species around FADs are typically mixed in nature [2,9–11]. Although skipjack tuna stocks are not currently considered to be overfished, concerns do exist for the on-going sustainability of bigeye and yellowfin tuna in the Pacific Ocean [12]. For some bigeye stocks, this incidental catch can contribute ~50% of the overall estimated fishery impact on the population [6]. There is therefore a need for the development of effective management measures that will reduce the fishing impact on juvenile bigeye and yellowfin stocks whilst maintaining maximum viability in purse seine fleets targeting skipjack. In the Pacific Ocean, periodic and regional closures of purse-seine fisheries [13,14] have been implemented to limit potential recruitment overfishing caused by catches of small bigeye tuna. Despite these measures, effort associated with FADs continues to increase [15–17].

The presence of floating objects is hypothesised to change the behaviour of tuna and other pelagic species [18–21], and in doing so may drive changes to tuna biology and ecosystem function in unknown ways when large numbers of man-made floating objects are introduced into the environment [21–24]. Floating objects are believed to attract tuna from up to 10km away [25,26], retain aggregations in the vicinity of the object [27–29], and alter their daytime vertical behaviour and habitat-use [19,30]. This alteration in habitat-use may also extend to changes in feeding behaviour, with some evidence that the trophic ecology of FAD-associated tuna is different to that of their free-swimming counterparts [31,32]. The time-series from archival electronic tags deployed in tuna also show extended shallow and surface-associated behaviour that may last several days [33,34] and studies have inferred that some of these observed associations are likely to be related to floating objects [18,34–37]. The prolonged periods of time in shallow water in the vicinity of floating objects increase the availability of tuna to surface fisheries such as purse-seine fishing fleets [34,38]. The combined effects of these behaviours in the presence of large numbers of introduced floating-objects have raised the concern that FADs may constitute an ecological trap, altering tuna’s perception of their environment and causing them to make potentially maladaptive habitat choices [21,39,40]. An improved understanding of the dynamic between tropical tunas and floating objects is needed for the development of effective management measures to conserve these species, particularly in regions of high concentrations of FAD utilisation [41,42].

In this study, we examine the vertical behaviour of bigeye and yellowfin tuna using a state-space modelling approach to depth and water temperature data collected by electronic archival tags deployed in fish throughout the WCPO. The number and length of periods of extended shallow surface-associations in the two species are examined in relation to when and where they occur to investigate potential linkages with the distribution of floating objects and implications for fisheries management.

## Materials and methods

### Data

Time-series data (summarised in the Supporting Information) were derived from archival tags deployed and recovered on 50 bigeye tuna captured whilst associated with anchored Tropical Atmosphere Ocean buoys or tagging vessels in the central equatorial Pacific (mean fork length 72cm, ±13.3 SD, Fig 1.), and 35 yellowfin tuna in the western Pacific whilst in a mixture of floating object-associated and free-schooling groups (mean fork length 70cm, ±14 SD, Fig 1.). All tag releases were undertaken as part of the Western and Central Pacific Fisheries Commission approved Pacific Tuna Tagging Programme [43]. Tagging of tuna in the Exclusive Economic Zone (EEZ) of Papua New Guinea and Kiribati was undertaken with additional permission from the respective Governments of each EEZ. Tagging at high seas locations did not require specific permissions. Tags deployed were a mixture of Lotek (St Johns, Canada) and Wildlife Computer (Redmond, USA) archival tags, which recorded light, water pressure (depth), internal body and ambient water temperature at intervals ranging from 10 to 300 seconds. Methods employed in the capture of fish and tag deployments were similar to those described in [44] and [19]. All tag releases were undertaken as part of the Western and Central Pacific Fisheries Commission approved Pacific Tuna Tagging Project. Tagging of tuna in the Exclusive Economic Zone (EEZ) of Papua New Guinea and Kiribati was undertaken with additional permission from the respective Governments of each EEZ.

Time-series consisted of at least 30 days of data that did not contain missing or corrupted data of greater than one hour, allowing adequate duration for model estimation [45]. Three time-series from bigeye tuna and four from yellowfin tuna were truncated due to tag malfunction and were therefore unable to be included in the examination of behaviour immediately prior to recapture, but are included in all other analyses. Time-series are publically available on request at http://www.spc.int/tagging/webtagging.

### Behavioural classification

Time-series were automatically classified using a multivariate-normal hidden Markov model (HMM), as described in [45]. Depth and water temperature measurements were firstly divided into three-hour time periods and the standard deviation of depth (diving amplitude) and mean water temperature (thermal habitat) calculated for each period (see Supporting Information for details on data preparation). These summary data were then arranged in a bivariate time-series, forming an observation model on which assumed two-state HMMs were estimated for each individual. Each HMM therefore provided a probabilistic classification of behaviour at each three-hour time-step into either relatively warm and shallow or relatively deep and cold behavioural states. Mean shallow state thermal habitat ranged from 24.0–29.0°C in bigeye, and 25.1–30.7°C in yellowfin tuna.

We assumed that continuous observations of shallow state classification over 24 hours represented a period of general surface-association behaviour by tuna, which is consistent with the potential floating object-association identified in previous studies [19,30,36,46]. The classification method also allowed quantification of the changing probability of this surface-association throughout each time-series. This was achieved by calculating a running mean of the probability of being in a relatively warm shallow state for each three-hour time-step, across a window of eight time-steps (24 hours). We refer to this running mean as the surface-association probability.

**Fig 2:**
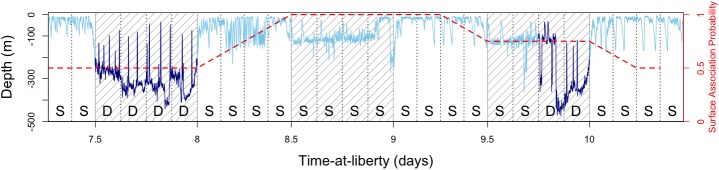
Example surface-association behaviour. Times series of depths experienced across three days from a tagged bigeye tuna. Day periods are identified as hatched with time divided into three hour periods (vertical dotted lines). Individual three-hour periods have been classified into either shallow (light blue and S) or deep (dark blue and D) state behaviours. The surface-association probability is shown in red.

Using this method, individual surface-association events can therefore be defined as periods in the time-series at which the surface-association probability exceeds a particular threshold value across concurrent time-steps. As an example, if an individual demonstrated the diel habitat switching behaviour that has typically been observed in both yellowfin and bigeye tunas [43–45], the calculated surface-association probability would be approximately 0.5 (Fig 2), as half of the running mean window would have a probability of 1 (12 hours of shallow behaviour) and the other half a probability of 0 (12 hours of deep behaviour). Greater time spent in warm shallow waters (such as during typical floating object association) would result in probabilities of surface-association closer to 1, while greater time in cooler, deeper waters (such as that which might occur during association with the thermocline) would result in probabilities of less than 0.5.

To examine the effect of different assumed threshold values on the number and length of surface-associations identified by our method, the proportion of each time-series defined as surface-associative was calculated for a range of thresholds. A threshold of 0.99 was associated with very few defined events, and 0.01 resulted in almost the entire time-series being identified as a surface-associative event. We visually examined proportion of the time-series identified as surface associative between this range of values in steps of 0.02. A value of 0.75 was chosen a consistent threshold used to define a single event, as proportion of time identified as surface-associative increased markedly for this value for all individuals (see Supporting Information).

To describe the occurrence of surface-association events identified from each individual, a number of summary metrics were calculated. These comprised: mean surface-association probability at the start, end and over the entirety of the time-series, and the proportion of the time-series exhibiting surface-association. The length of first, last and all surface-associations combined were examined, alongside the mean number of surface-associations per month-at-liberty. The surface-association probability over each day of the final week at liberty was also plotted. All results were separated by species.

### Light-based geolocation

A subset of 20 time-series collected from yellowfin tuna tagged in the Bismarck Sea and Solomon Sea region was examined for spatial linkages between surface associated behaviour and regions of high concentrations of FADs. An equivalent analysis for bigeye was not conducted due to low spatial overlap. Horizontal positions of individuals were estimated by fitting a modified state-space model *trackit* [47] to time-series of light data. Confidence intervals at 95% associated with each position estimate were calculated to provide a measure of the potential spatial position of each fish at three time-points per day.

#### Combining horizontal movement and surface-association behaviour

The position estimate at each time-step was represented as an ellipse, with major and minor axes equal to the 95% confidence interval in longitudinal and latitudinal dimensions. Each ellipse was assigned the surface-association probability from the closest corresponding three-hour time-bin of vertical behaviour. The ellipses were then rasterised to polygons (discretised) at 0.1 degree cell resolution using Quantum GIS [48], with each raster cell assigned the same value of surface-association probability that was held by the original ellipse.

The raster cells of each polygon from all geolocated tracks were combined into a single raster map of cells. Where raster cells from multiple polygons overlapped, the mean of the surface-association probability of all the individual cells was assigned to the map cell, assuming independence of observation for each geolocation point regardless of the number of individual fish the location was drawn from. The resulting map was therefore a 0.1 degree cell resolution raster grid, with each cell providing the mean surface-association probability from fish that were potentially present in that cell. An example geolocation time-series is given, alongside a visual representation of how multiple tags were combined, in the Supporting Information.

Areas of consistently high or low surface associated behaviour were visually identified from the raster map and then compared with the spatial distribution of known floating-object associated purse seine sets that occurred during the period in which the tags were deployed (2006–2014) using catch data available through the Western and Central Pacific Fisheries Commission.

## Results

### Surface-association events at release

**Fig 3:**
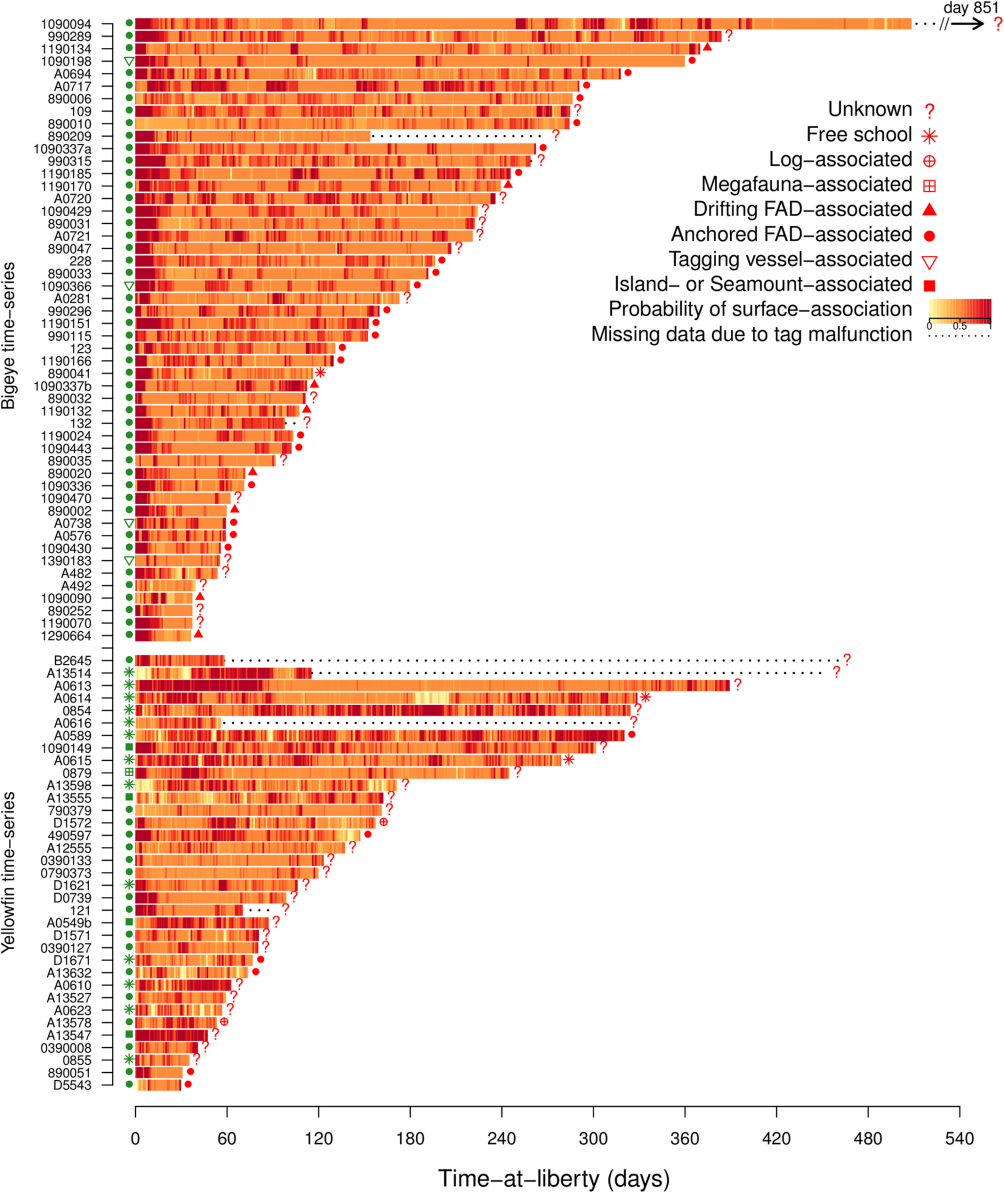
Surface-association time-series. Surface-association probabilities through time, calculated for bigeye (top) and yellowfin (bottom) tuna tagged in the western and central Pacific Ocean. Behaviour of the school from which tagged individuals were derived at release and recapture are shown at the beginning and end of the time-series, respectively.

**Fig 4:**
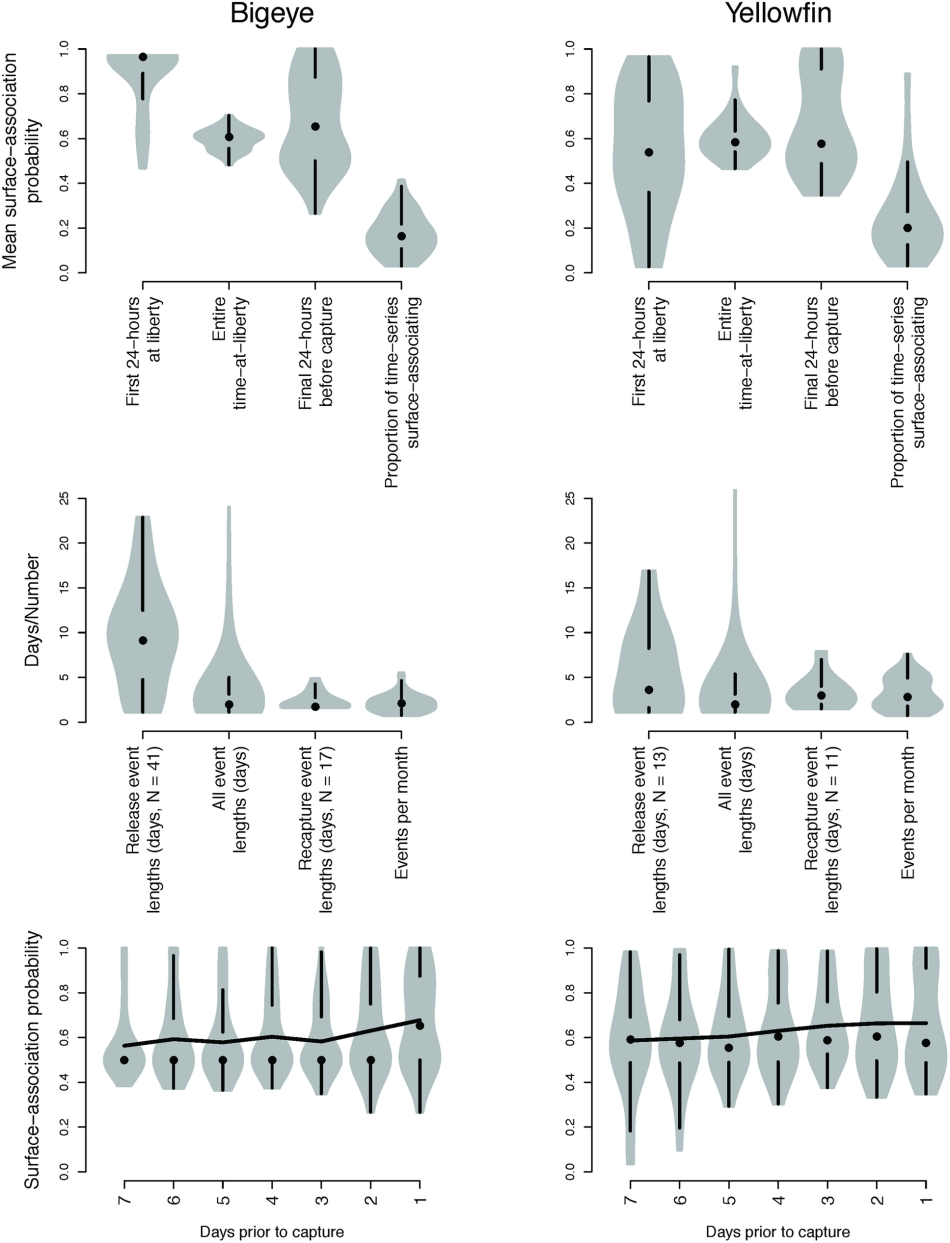
Surface-association summary metrics. Violin plots of surface association probability summary metrics for bigeye (left panel) and yellowfin (right panel) tuna, assuming a probability threshold of 0.75 for classification.

Surface-association events occurred within the 24 hours immediately following tagging in 82% of bigeye tuna and 37% of yellowfin tuna (Fig 3). Surface-association events during this period were longer than the median event length, and in 62% of bigeye tuna and 17% of yellowfin tuna, represented the longest periods of surface-association in the whole time-series. Of those bigeye that demonstrated surface-association behaviour in the 24 hours immediately after release, the mean duration was 9.6 days (SD ±5.75). Three bigeye individuals exhibited surface-associations of more than 20 days. The high incidence of this behaviour was also reflected in consistently high surface-association probability during the first 24-hours after release (Fig 4).

The mean length of surface-association events immediately after release recorded by tagged yellowfin tuna was 5.7 (SD ±4.9) days, across 13 fish. Surface-association events immediately after release were predominantly demonstrated by fish caught and tagged associated with FADs, as well as two free schooling individuals, and one megafauna- and one seamount-associated fish. This behaviour by FAD-associated fish lasted less than 2.5 days for six fish, and 8.25 to 11.25 days for the remaining three. For the two free schooling fish, surface-associations were of 3.6 and 7.9 days duration.

### Surface-association throughout time-at-liberty

Surface-association events in bigeye tuna were often interspersed between sustained periods of lower surface-association probabilities of around 0.5, characteristic of diel switching behaviour between deeper cooler waters during the day and shallower, warmer waters during the night (Fig 3). Overall, the total time spent demonstrating surface-associated behaviour was low with tagged bigeye spending on average 17% (±1.3 s.e., 11% when excluding any surface-association immediately after release) of time demonstrating this behaviour (Fig 4). Surface-association events identified throughout the time-series were generally short, with a median of two days (1.75 days IQR) and a maximum of 24 days (Fig 4). A number of individual fish demonstrated several short surface-association events in succession throughout a time-series (e.g. tags 228; 980006). Overall the mean number of events per month was 2.1.

Periods of surface-association demonstrated by yellowfin tuna were similar to those in bigeye tuna, with a median a two days (1.5 days IQR) and a maximum of 28.1 days. Overall, the average total time spent demonstrating surface-associated behaviour was 23% of the time at liberty (±2.9 s.e., 22% when excluding any surface-association immediately after release) (Fig 4). A small number of yellowfin exhibited very long periods of near constant surface-association (e.g. A0854; A0613) with one yellowfin tuna exhibiting over 80 days beginning around three days after release in a free school (Fig 3). The overall mean number of events was 3.2 events per month, with one individual demonstrating on average more than 7.5 events per month (Fig 4).

The length of individual surface-association events was positively skewed towards longer associations for both species, even when removing any, typically long, release event associations. To identify unusually long surface-associations, we used a measure of significant deviation from the central spread of event lengths to be 1.5 times the interquartile range greater than the upper quartile. Using this method, 11.9% of all bigeye surface-association events were outside the central spread of lengths (9.1% excluding any release events), and such events were exhibited by 41 of the 50 individuals. For yellowfin, 8.6% of surface-association events were outside the central spread of event lengths (7.8%, excluding any release events), exhibited by 18 of the 35 individuals.

### Surface-association events at recapture

Of these individuals where time-series were complete to recapture (bigeye tuna: n = 47; yellowfin tuna: n = 31), surface-associations were detected in the period immediately prior to recapture in 17 bigeye (36%) and 10 yellowfin tuna (32%) (Table 1). The remainder showed no signs of surface-association prior to recapture. As well as being less frequently demonstrated by tagged fish, surface-associations at recapture were much shorter than those immediately after release associations. The mean probability of surface-association during the 24-hours prior to recapture was bimodal for bigeye (Hartigan’s dip test of bimodality p = 0.004, Fig 4). The majority of individuals exhibited surface-association probabilities during this period comparable to the mean of the entire time-series, and a smaller group exhibited much higher surface-association probabilities of 80–90%. The duration of surface-association events prior to recapture were predominantly less than two days with a median of 1.75 days and a maximum of five days (Fig 4). Surface-association events prior to recapture were generally slightly longer and more normally distributed in tagged yellowfin tuna with a median of three days and a maximum of eight days. The distribution of surface-association probability approached bimodality during the final 24 hours for this species, however this was not statistically significant (Hartigan’s dip test of bimodality p = 0.08, Fig 4).

**Table 1:** Surface-association across set-type.

Species	Free school	Log-associated	FAD-associated	Unknown
Bigeye	0% (0/1)	N/A	41% (12/29)	29% (5/17)
Yellowfin	50% (1/2)	0% (0/2)	33% (2/6)	33% (7/21)

Proportions of tuna exhibiting a surface-association probability greater than 75% immediately prior to recapture, by set type.

### Spatial patterns

**Fig 5:**
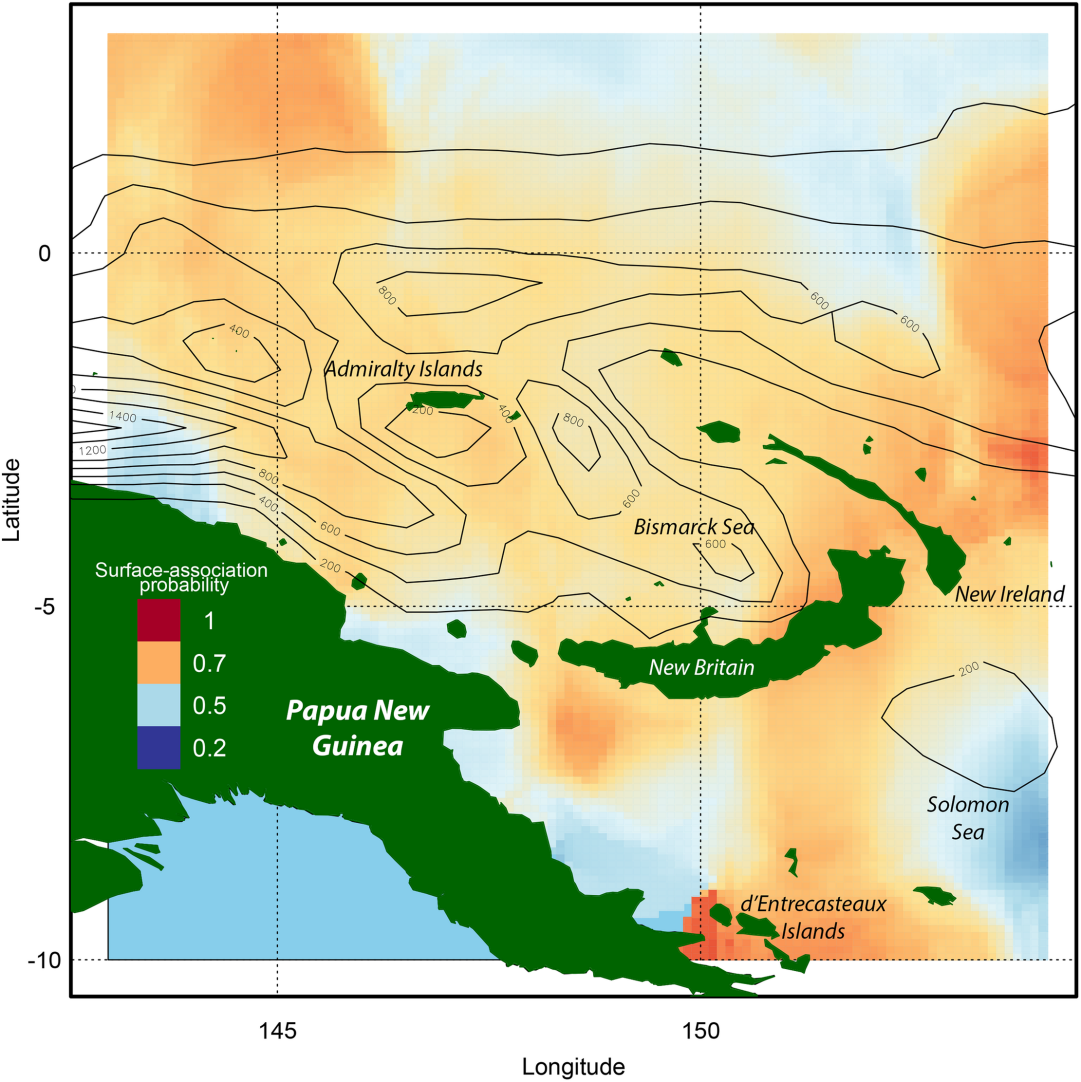
Spatial patterns of surface-association. The spatial distribution of surface-association probabilities estimated from 20 yellowfin tuna in the Bismarck and Solomon Sea region. Overlaid contours represent the number of floating object-associated sets recorded during the period 2006–2014 across the region.

Consistently high occurrence of surface-associated behaviour in the subset of yellowfin tuna examined occurred around the north west of the Bismarck Sea, the central region and eastern region around New Britain and New Ireland, and the area of the d’Entrecasteaux Islands, although in the latter region there were observations from only low numbers of individuals (Fig 5). Low surface-association probabilities occurred in the Solomon Sea area, and less consistently in the region north of the Bismarck Sea and in the EEZ of the Federated States of Micronesia. A north-south strip of relatively low surface-association probability was present in the gulf between the Admiralty Islands and New Ireland leading to the oceanic waters north of the Bismarck Archipelago.

Surface-association probability tended towards 0.5, representative of switching behaviour between shallower and deeper water, in the region to the north of the Bismarck Sea. The high surface-association probabilities exhibited around the island masses corresponded to some of the lowest densities of FAD-associated purse-seine sets recorded over the same period. Similarly, there was an apparent inverse association between the increase in the number of FAD sets within the deeper waters of the Solomon Sea and the prominence of surface-association. No spatial analysis was carried out for bigeye tuna in our study due to low geographic overlap across individuals.

## Discussion

In this study we have shown that surface-association behaviour in a moderate sample of electronically tagged bigeye and yellowfin tunas is highly variable through time for an individual, between individuals, and spatially in yellowfin tuna across regions with varying FAD densities. Surface-association events at release were inconsistent, but when present were generally sustained for longer periods than other events identified in the time-series. The high incidence of surface-associated behaviour immediately after release observed in bigeye was not surprising given that all bigeye tuna were caught for tagging whilst associated with floating objects, and the length of these events was consistent with the known association behaviour determined from acoustic tagging [18,19]. These mean surface-association durations immediately after release were similar to observations of FAD-residence reported in the north-western Pacific (mean = 8.3 days; [28]), and longer than those observed in the Bismarck Sea and Solomon Sea (approximately 4 days; [19]).

This higher occurrence and longer duration of surface-association behaviour immediately after release may be reflective of the trauma associated with capture, handling and tagging. Handling induced behavioural modification has been reported in a number of pelagic species and can manifest as decreased vertical activity, although there are few documented changes in behaviour reported from tunas [49,50]. It should be noted that such assessments of behaviour in tunas to date have only been conducted on summarised archival tag data and from large individuals. Smaller scale changes in behaviour may not be able to be determined from summarised data [50], and larger individuals may not be impacted by capture, handling and tagging in the same way as smaller fish. Association with floating objects has also been hypothesised to be a mechanism in school formation and conspecific mixing, as suggested by the meeting point hypothesis [51]. Mild trauma brought on by handling may negatively impact participation by an individual in normal school formation and subsequent movement of that school away from a floating object. Longer residence times would result while the fish first recovers and then subsequently joins another school after it forms over a period of time.

There are a number of potential explanations for the shorter surface-behaviour patterns of yellowfin following tagging release, when compared to patterns observed in our sample of bigeye. Surface- or floating object-association behaviour could occur in a fundamentally different way in yellowfin tuna, although this seems unlikely given previous observations of this species when known to be in the vicinity of floating objects [19,30]. If trauma does occur, it may have less of an obvious impact, or is not pronounced enough for the classification framework applied here to discern. Differences in the vertical behaviour between the two species may also result in the classification method being unable to identify shallow, warm behaviour as robustly in yellowfin as in bigeye. However, as behavioural HMMs were estimated from each time-series individually, it is unlikely that we would systematically fail to identify relatively shallow behaviour for yellowfin alone. Spatial differences in the biotic or abiotic environment between oceanic and archipelagic environments may have a greater influence on differences in post tagging behaviour than a species-specific trauma effect. Of note is that, of those yellowfin that demonstrated prolonged surface-association events lasting longer than 2 days when released at a FAD (N = 4), three of those fish were released in the central equatorial Pacific (tags 121; D0739 and 490597, Fig 3). Residence times of tagged yellowfin tuna immediately after release around FADs reported elsewhere vary, with yellowfin tuna in Hawaiian waters resident for 8.0 ± 12.6 days [28] and, for previous experiments from our study region, less than 21 hours and up to 15 days [19].

For the surface-association events throughout time-at-liberty, our estimate of surface associative behaviour for yellowfin was considerably lower than for fish believed to be associating with FADs in previous studies, which spent up to 60% [27] and 64% [52] of their time at the surface. Our results for both species are closer to those reported for bigeye in the north-western Pacific (13%; [36]), in the equatorial eastern Pacific (9%-19%, [34]), and yellowfin (10%) and bigeye (16%) in the equatorial eastern Pacific [30].

Our results suggest that bigeye and yellowfin tuna may demonstrate two different behavioural modes of surface-association during time-at-liberty: a more frequent short duration association, and a less frequent extended duration association with the surface layer [53]. It is possible that some of these short surface-associations may reflect behaviours that are completely independent of floating-object association, such as periods of active horizontal relocation to new areas [54], spawning [33], opportunistic feeding [31] or some other unknown behaviour. Fish briefly visiting floating objects, travelling between FADs in an array, or associating non-continuously with frequent excursions away from the device have also been suggested in a number of previous studies that have described associative behaviour in tuna within FAD arrays near island masses [19,28,55].

In the days leading up to recapture, considerable individual variation in surface-association probabilities was identified in both species. Although a number of tagged fish were identified as being caught whilst associated with floating objects (62% of bigeye, 26% of yellowfin), less than half exhibited surface-association in the period immediately prior to recapture (Fig 4). In the majority of cases, it either appears that arrival at a floating object coincided with, or was just prior to, recapture, or that tuna do not always exhibit consistent surface-associated behaviours when associated with a floating object.

When examining our spatial patterns of surface-association in yellowfin, our method of combining the two-dimensional surfaces of 95% confidence interval location aimed to identify the clearest patterns of behaviour. However, as there is considerable error around light-based geolocation estimates for tuna species in equatorial regions [47,56,57], care must be taken when considering latitudinal areas of less than 2 degrees. Despite this, greater probability of surface-association was consistently estimated around the island masses surrounding the Bismarck Sea, where bathymetry, biotic environment and density of natural floating objects is likely to be different to more oceanic waters to the North and East. The anchored FADs in the Bismarck Sea and coastal Solomon Islands reside in a region of dominated by archipelagic waters, islands and seamounts, and this habitat may impact the motivations behind FAD-association [6,58]. Although the true local stimulus experienced by these individual fish can never be known, it could be hypothesised that coastal- or island- association behaviours reflect different prey sources, such as fish larvae and juveniles that aggregate near the surface [23,32], and appear similar in depth and temperature patterns to those of FAD-associative behaviour. It is likely that local prey availability may induce greater periods of time near the surface, irrespective of floating object density.

We propose that processes working at different scales may explain the inter- and intra-individual variability in surface behaviours observed here. At the finest scale, the availability of prey, the local density of conspecifics, and the multi-species composition of the schools themselves, are responsible for many of the temporal dynamics in tuna behaviour generally [29,59,60]. The use of FADs by tuna is also likely to be the result of the complex interactions between these local environmental drivers and the at-present unclear drivers of attraction and aggregation around floating objects [6,22]. Small variations in local interactions can lead to significant variation in emergent behaviours at larger scales. It is possible that floating objects contribute to the concentration of schools horizontally at local scales, whilst islands and other bathymetric features may affect vertical behaviour at larger spatial scales.

Purse-seiners set on floating objects because they bring tuna to a more easily found locality in horizontal space, and then aggregate them in relative shallow water through this surface behaviour. The surface-association events we have identified in this study vary greatly. While some events are clear and prolonged, the large majority are not, and extended surface-association behaviour is rarely exhibited immediately prior to capture.

### Fisheries implications

It must be stressed that the size class of our study fish overlaps with that of only the larger bigeye and yellowfin individuals typically caught in floating object-associated purse seine sets [2] (Fig 1), and there may be a ontogenetic propensity for surface-association behaviours that changes with age and size, such as the development of the swim-bladder [61]. However, for the size class of individuals examined here, we suggest that there is little evidence of sustained and consistent periods of time near the surface during which fish are more vulnerable to purse seine capture, even in regions where FAD deployment is common. For the majority of the fish we have examined here, it appears that one instance of many common short-term residences around a floating object resulted in capture. This supports the proposal that if the large majority of residences at floating objects by tuna are moderately short, then there is little evidence to suggest that their biology, movement behaviours or entrainment to a region are being significantly affected (34). If long periods of entrainment near the surface at floating object by tuna do not occur regularly, then it is the concentration of fish over short periods in the horizontal plane that increases the probability of fish being captured.

Current management measures in large-scale tuna fisheries, such as the WCPO, have restricted FAD use in certain regions and for periods of time [14]. However analyses of these measures shows that they simply shift effort on FADs from one place or time to another with less than anticipated impacts on the overall catch or the number of sets made [16]. If FADs are not retaining fish in specific areas but rather increase the efficiency for locating tuna schools suitable for fishing, then the effectiveness of management measures restricting the overall effort on floating objects should be examined further. Current management measures, which invoke the spatial closure of certain areas in order to restrict the catch of surface gears, may only be beneficial if they co-occur within regions where large-scale archipelagic processes are less present and where fish are not already exhibiting surface-association behaviours. Furthermore, understanding surface-associated behaviour in this context highlights the need to explore new and alternative tagging technologies, which will allow data on this behaviour to be captured for the small to medium size-classes of tropical tuna most vulnerable to purse seine capture.

## Conclusion

Our methodological approach demonstrates the benefits of classification of telemetry data using state-space models with an observation model constructed to answer specific behavioural questions. As increasing data on the environmental context of tunas becomes available, such as through FAD-installed acoustic receivers and echo sounders, this covariate information should be incorporated into the parameter estimation. Both behavioural state distribution and state-switching parameters may change in relation to these variables [45,62], and their effect on the occurrence of surface-association should be examined. While this state-space classification method identifies relative shallow and deep states that may vary between individuals, this analysis does not specifically consider the differences between shallow and deep behavioural states themselves. This variation in depth across individuals and between these species should be examined in the broader of context of yellowfin and bigeye tuna behaviour, particularly across the size classes most caught by purse seine gears.

However, without direct observation of the presence of FADs, and the occurrence and dynamics of the local tuna-prey environment, identifying drivers for behaviour around floating objects will always be a challenge. The instrumentation of FADs [63] provides an opportunity to collect this information. Industry-deployed smart-FADs, that can record information on drifting FAD movement, local environmental conditions, the biomass of local tuna aggregations, and potentially house receivers for acoustic tags deployed on tunas, have the potential to create an ocean network of autonomous observation stations [64,65].

## Supporting information

S1 File **Table A in S1 file. Summary of electronic tagging data used in this analysis**.
**Figure A in S1 file. Varying surface-association threshold probability.** Proportion of all time-series classified as “surface-associative”, by assumed threshold surface-association probability used in classification. Data are shown as simplified boxplots, with outliers of more than 1.5 the interquartile range plotted as hollow circles.
**Figure B in S1 file. Conceptual diagram of combined horizontal and vertical behaviour.** Diagram showing the combination of horizontal geolocation error and surface-association probability. An example time-series is shown on the left, from which geolocation polygons are rasterised and combined with time-series from other tags. The surface-association probability of the example raster map cell is an equally weighted mean of A1, A2, B1 and B2.
(DOCX)
