# Supplementary material for: Revisiting the vulnerability of juvenile bigeye (Thunnus obesus) and yellowfin (T. albacares) tuna caught by purse-seine fisheries while associating with surface waters and floating objects
Source: PLoS One. 2017 Jun 29;12(6):e0179045. doi: 10.1371/journal.pone.0179045 (PMC5490998; doi:10.1371/journal.pone.0179045)
Supplement: S1 File — Table A in S1 file. Summary of electronic tagging data used in this analysis. Figure A in S1 file. Varying surface-association threshold probability. Proportion of all time-series classified as “surface-associative”, by assumed threshold surface-association probability used in classification. Data are shown as simplified boxplots, with outliers of more than 1.5 the interquartile range plotted as hollow circles. Figure B in S1 file. Conceptual diagram of combined horizontal and vertical behaviour. Diagram showing the combination of horizontal geolocation error and surface-association probability. An example time-series is shown on the left, from which geolocation polygons are rasterised and combined with time-series from other tags. The surface-association probability of the example raster map cell is an equally weighted mean of A1, A2, B1 and B2. (DOCX) [file pone.0179045.s001.docx]

Surface-associated Behaviours of Bigeye (*Thunnus obesus*) and Yellowfin (*T. albacares*) Tuna in the Western and Central Pacific Ocean

Joe Scutt Phillips, Graham M. Pilling, Bruno Leroy, Karen Evans, Thomas Usu, Tim Lam, Kurt M. Schaefer & Simon Nicol

S1 Supporting Information

*Time-series Data*

Table A

| **TagID** | **Species** | **Fork Length at Release (cm)** | **Tag Manufacturer and Model** | **Release Date** | **Release Latitude** | **Release Longitude** | **School Behaviour at Release** | **Recording Interval (seconds)** | **Recapture Date** | **Time-at-liberty (days)** | **School Behaviour at Recapture** |
| --- | --- | --- | --- | --- | --- | --- | --- | --- | --- | --- | --- |
| 1290664 | Bigeye | 82 | Wildlife Computers - MK9 | 23/11/13 | -2.095 | 189.983 | Anchored FAD | 30 | 30/12/13 | 37 | Drifting FAD |
| 1190070 | Bigeye | 69 | Wildlife Computers - MK9 | 22/11/13 | -2.093 | 189.985 | Anchored FAD | 30 | 30/12/13 | 38 | Unknown |
| 890252 | Bigeye | 82 | Wildlife Computers - MK9 | 12/10/09 | 0.010 | 205.410 | Anchored FAD | 30 | 19/11/09 | 38 | Unknown |
| 1090090 | Bigeye | 85 | Wildlife Computers - MK9 | 13/05/10 | 2.008 | 189.981 | Anchored FAD | 30 | 20/06/10 | 38 | Drifting FAD |
| A492 | Bigeye | 48 | Lotek - L25 | 27/03/09 | -0.010 | 155.576 | Anchored FAD | 240 | 06/05/09 | 40 | Unknown |
| A482 | Bigeye | 52 | Lotek - L25 | 26/03/09 | -1.590 | 156.021 | Anchored FAD | 240 | 20/05/09 | 55 | Unknown |
| 1390183 | Bigeye | 81 | Wildlife Computers - MK9 | 25/11/13 | -0.150 | 189.735 | Tagging vessel-associated | 30 | 20/01/14 | 56 | Unknown |
| 1090430 | Bigeye | 72 | Wildlife Computers - MK9 | 14/10/11 | -0.015 | 189.977 | Anchored FAD | 30 | 10/12/11 | 57 | Anchored FAD |
| A0576 | Bigeye | 51 | Lotek - L28 | 05/12/11 | 8.002 | 204.995 | Anchored FAD | 30 | 03/02/12 | 60 | Anchored FAD |
| A0738 | Bigeye | 51 | Lotek - L28 | 10/12/11 | 7.585 | 204.981 | Tagging vessel-associated | 30 | 08/02/12 | 60 | Anchored FAD |
| 890002 | Bigeye | 68 | Wildlife Computers - MK9 | 11/05/08 | 5.017 | 205.432 | Anchored FAD | 30 | 11/07/08 | 61 | Drifting FAD |
| 1090470 | Bigeye | 78 | Wildlife Computers - MK9 | 12/10/11 | -0.011 | 189.977 | Anchored FAD | 30 | 14/12/11 | 63 | Unknown |
| 1090336 | Bigeye | 80 | Wildlife Computers - MK9 | 24/11/10 | -0.020 | 189.965 | Anchored FAD | 30 | 04/02/11 | 72 | Anchored FAD |
| 890020 | Bigeye | 70 | Wildlife Computers - MK9 | 16/05/08 | 1.595 | 205.426 | Anchored FAD | 30 | 28/07/08 | 73 | Drifting FAD |
| 890035 | Bigeye | 106 | Wildlife Computers - MK9 | 18/05/08 | 1.595 | 205.425 | Anchored FAD | 30 | 19/08/08 | 93 | Unknown |
| 1090443 | Bigeye | 77 | Wildlife Computers - MK9 | 11/10/11 | -0.011 | 189.977 | Anchored FAD | 30 | 22/01/12 | 103 | Anchored FAD |
| 1190024 | Bigeye | 76 | Wildlife Computers - MK9 | 12/10/11 | -0.011 | 189.977 | Anchored FAD | 30 | 24/01/12 | 104 | Anchored FAD |
| 132 | Bigeye | 60 | Lotek - L28 | 27/10/09 | 1.585 | 219.992 | Anchored FAD | 60 | 03/02/10 | 108 | Unknown |
| 1190132 | Bigeye | 67 | Wildlife Computers - MK9 | 03/10/12 | -0.031 | 189.973 | Anchored FAD | 30 | 19/01/13 | 108 | Drifting FAD |
| 890032 | Bigeye | 86 | Wildlife Computers - MK9 | 17/05/08 | 1.591 | 205.421 | Anchored FAD | 30 | 06/09/08 | 112 | Unknown |
| 1090337b | Bigeye | 61 | Wildlife Computers - MK9 | 01/10/12 | -2.090 | 189.980 | Anchored FAD | 30 | 22/01/13 | 113 | Drifting FAD |
| 890041 | Bigeye | 79 | Wildlife Computers - MK9 | 24/05/08 | 1.594 | 205.425 | Anchored FAD | 30 | 18/09/08 | 117 | Unassociated / Free school |
| 1190166 | Bigeye | 76 | Wildlife Computers - MK9 | 13/11/11 | 0.009 | 220.451 | Anchored FAD | 30 | 23/03/12 | 130 | Anchored FAD |
| 123 | Bigeye | 59 | Lotek - L28 | 27/10/09 | 1.585 | 219.992 | Anchored FAD | 60 | 08/03/10 | 131 | Anchored FAD |
| 990115 | Bigeye | 58 | Wildlife Computers - MK9 | 26/05/09 | 0.004 | 206.471 | Anchored FAD | 30 | 26/10/09 | 153 | Anchored FAD |
| 1190151 | Bigeye | 67 | Wildlife Computers - MK9 | 13/11/11 | 0.009 | 220.451 | Anchored FAD | 30 | 15/04/12 | 153 | Anchored FAD |
| 990296 | Bigeye | 72 | Wildlife Computers - MK9 | 26/10/09 | 1.585 | 219.992 | Anchored FAD | 30 | 05/04/10 | 161 | Anchored FAD |
| A0281 | Bigeye | 59 | Lotek - L25 | 08/11/08 | -0.205 | 147.379 | Anchored FAD | 240 | 01/05/09 | 174 | Unknown |
| 1090366 | Bigeye | 96 | Wildlife Computers - MK9 | 23/11/10 | -0.018 | 189.900 | Tagging vessel-associated | 30 | 23/05/11 | 180 | Anchored FAD |
| 890033 | Bigeye | 85 | Wildlife Computers - MK9 | 17/05/08 | 2.000 | 205.000 | Anchored FAD | 30 | 26/11/08 | 193 | Anchored FAD |
| 228 | Bigeye | 61 | Lotek - L28 | 27/10/09 | 1.585 | 219.992 | Anchored FAD | 60 | 12/05/10 | 197 | Anchored FAD |
| 890047 | Bigeye | 83 | Wildlife Computers - MK9 | 24/05/08 | 1.594 | 205.425 | Anchored FAD | 30 | 18/12/08 | 208 | Unknown |
| A0721 | Bigeye | 89 | Lotek - L28 | 14/11/11 | -0.015 | 220.452 | Anchored FAD | 30 | 23/06/12 | 221 | Unknown |
| 890031 | Bigeye | 77 | Wildlife Computers - MK9 | 17/05/08 | 1.591 | 205.421 | Anchored FAD | 30 | 26/12/08 | 223 | Unknown |
| 1090429 | Bigeye | 68 | Wildlife Computers - MK9 | 19/10/11 | -0.010 | 180.460 | Anchored FAD | 30 | 31/05/12 | 225 | Unknown |
| A0720 | Bigeye | 51 | Lotek - L28 | 07/12/11 | 7.512 | 205.415 | Anchored FAD | 30 | 31/07/12 | 236 | Unknown |
| 1190170 | Bigeye | 70 | Wildlife Computers - MK9 | 20/10/11 | -0.013 | 180.455 | Anchored FAD | 30 | 16/06/12 | 240 | Drifting FAD |
| 1190185 | Bigeye | 79 | Wildlife Computers - MK9 | 13/11/11 | 0.009 | 220.451 | Anchored FAD | 30 | 17/07/12 | 247 | Anchored FAD |
| 990315 | Bigeye | 63 | Wildlife Computers - MK9 | 26/10/09 | 1.585 | 219.992 | Anchored FAD | 30 | 15/07/10 | 262 | Unknown |
| 1090337a | Bigeye | 84 | Wildlife Computers - MK9 | 26/11/10 | 2.011 | 189.983 | Anchored FAD | 30 | 16/08/11 | 263 | Anchored FAD |
| 890209 | Bigeye | 62 | Wildlife Computers - MK9 | 05/11/08 | 0.159 | 145.481 | Anchored FAD | 30 | 09/04/09 | 271 | Unknown |
| 890010 | Bigeye | 72 | Wildlife Computers - MK9 | 16/05/08 | 1.591 | 205.423 | Anchored FAD | 30 | 25/02/09 | 285 | Anchored FAD |
| 109 | Bigeye | 68 | Lotek - L28 | 27/10/09 | 1.585 | 219.992 | Anchored FAD | 60 | 09/08/10 | 285 | Unknown |
| 890006 | Bigeye | 73 | Wildlife Computers - MK9 | 16/05/08 | 1.591 | 205.423 | Anchored FAD | 30 | 27/02/09 | 287 | Anchored FAD |
| A0717 | Bigeye | 69 | Lotek - L28 | 14/11/11 | -0.015 | 220.452 | Anchored FAD | 30 | 01/09/12 | 291 | Anchored FAD |
| A0694 | Bigeye | 53 | Lotek - L28 | 09/12/11 | 7.571 | 204.998 | Anchored FAD | 30 | 23/10/12 | 318 | Anchored FAD |
| 1090198 | Bigeye | 106 | Wildlife Computers - MK9 | 24/11/10 | -0.018 | 189.900 | Tagging vessel-associated | 30 | 20/11/11 | 361 | Anchored FAD |
| 1190134 | Bigeye | 69 | Wildlife Computers - MK9 | 03/10/12 | -0.032 | 189.973 | Anchored FAD | 30 | 09/10/13 | 371 | Drifting FAD |
| 990289 | Bigeye | 69 | Wildlife Computers - MK9 | 26/10/09 | 1.585 | 219.992 | Anchored FAD | 30 | 15/11/10 | 385 | Unknown |
| 1090094 | Bigeye | 87 | Wildlife Computers - MK9 | 27/11/10 | 2.011 | 189.983 | Anchored FAD | 30 | 19/04/12 | 851 | Unknown |
| D5543 | Yellowfin | 56 | Lotek - L23 | 05/08/09 | -2.499 | 150.407 | Anchored FAD | 60 | 04/09/09 | 30 | Anchored FAD |
| 890051 | Yellowfin | 88 | Wildlife Computers - MK9 | 01/06/08 | 17.122 | 202.526 | Anchored FAD | 30 | 03/07/08 | 32 | Anchored FAD |
| 855 | Yellowfin | 58 | Lotek - L28 | 18/04/13 | -6.038 | 151.181 | Unassociated / Free school | 10 | 24/05/13 | 36 | Unknown |
| 390008 | Yellowfin | 104 | Wildlife Computers - MK9 | 05/09/06 | -3.406 | 149.502 | Anchored FAD | 60 | 17/10/06 | 42 | Unknown |
| A13547 | Yellowfin | 68 | Lotek - L24 | 20/09/06 | -2.568 | 145.074 | Seamount-associated | 300 | 07/11/06 | 48 | Unknown |
| A13578 | Yellowfin | 55 | Lotek - L24 | 21/09/06 | -3.502 | 145.342 | Anchored FAD | 300 | 14/11/06 | 54 | Log |
| A0623 | Yellowfin | 63 | Lotek - L28 | 29/01/12 | -6.166 | 150.485 | Unassociated / Free school | 10 | 27/03/12 | 58 | Unknown |
| A13527 | Yellowfin | 71 | Lotek - L24 | 20/09/06 | -2.396 | 145.051 | Anchored FAD | 300 | 19/11/06 | 60 | Unknown |
| A0610 | Yellowfin | 61 | Lotek - L28 | 28/01/12 | -6.124 | 150.596 | Unassociated / Free school | 10 | 01/04/12 | 64 | Unknown |
| A13632 | Yellowfin | 55 | Lotek - L24 | 26/03/07 | -5.128 | 149.174 | Anchored FAD | 300 | 09/06/07 | 75 | Anchored FAD |
| D1671 | Yellowfin | 86 | Lotek - L23 | 15/04/07 | -2.551 | 150.431 | Unassociated / Free school | 60 | 02/07/07 | 78 | Anchored FAD |
| 390127 | Yellowfin | 109 | Wildlife Computers - MK9 | 19/09/06 | -2.396 | 145.051 | Anchored FAD | 60 | 09/12/06 | 81 | Unknown |
| D1571 | Yellowfin | 72 | Lotek - L23 | 27/03/07 | -5.133 | 149.171 | Anchored FAD | 60 | 16/06/07 | 81 | Unknown |
| A0549b | Yellowfin | 55 | Lotek - L28 | 29/05/11 | -1.445 | 146.116 | Seamount-associated | 240 | 25/08/11 | 88 | Unknown |
| 121 | Yellowfin | 78 | Lotek - L28 | 24/10/09 | -2.020 | 219.990 | Anchored FAD | 60 | 03/01/10 | 94 | Unknown |
| D0739 | Yellowfin | 68 | Lotek - L23 | 23/10/09 | -2.024 | 219.990 | Anchored FAD | 60 | 31/01/10 | 100 | Unknown |
| D1621 | Yellowfin | 86 | Lotek - L23 | 15/04/07 | -2.576 | 150.408 | Unassociated / Free school | 60 | 31/07/07 | 107 | Unknown |
| 790373 | Yellowfin | 69 | Wildlife Computers - MK9 | 27/03/08 | -8.400 | 158.566 | Anchored FAD | 60 | 26/07/08 | 121 | Other |
| 390133 | Yellowfin | 98 | Wildlife Computers - MK9 | 24/09/06 | -4.205 | 146.263 | Anchored FAD | 60 | 26/01/07 | 124 | Unknown |
| A12555 | Yellowfin | 77 | Lotek - L24 | 19/05/05 | -3.570 | 150.360 | Anchored FAD | 300 | 04/10/05 | 138 | Unknown |
| 490597 | Yellowfin | 68 | Wildlife Computers - MK9 | 10/05/08 | 7.579 | 205.408 | Anchored FAD | 30 | 05/10/08 | 148 | Anchored FAD |
| D1572 | Yellowfin | 75 | Lotek - L23 | 12/04/07 | -3.437 | 148.205 | Anchored FAD | 60 | 17/09/07 | 158 | Log |
| 790379 | Yellowfin | 62 | Wildlife Computers - MK9 | 27/03/08 | -8.392 | 158.376 | Anchored FAD | 60 | 05/09/08 | 162 | Unknown |
| A13555 | Yellowfin | 60 | Lotek - L24 | 20/09/06 | -2.568 | 145.074 | Seamount-associated | 300 | 02/03/07 | 163 | Unknown |
| A13598 | Yellowfin | 54 | Lotek - L24 | 13/03/07 | -1.385 | 150.442 | Unassociated / Free school | 300 | 01/09/07 | 172 | Unknown |
| 879 | Yellowfin | 71 | Lotek - L28 | 20/04/13 | -6.415 | 150.557 | Megafauna-associated | 10 | 22/12/13 | 246 | Unknown |
| A0615 | Yellowfin | 63 | Lotek - L28 | 29/01/12 | -6.166 | 150.485 | Unassociated / Free school | 10 | 04/11/12 | 280 | Unassociated / Free school |
| 1090149 | Yellowfin | 79 | Wildlife Computers - MK9 | 05/06/10 | 5.529 | 197.889 | Island-associated | 30 | 03/04/11 | 302 | Unknown |
| A0589 | Yellowfin | 51 | Lotek - L28 | 25/04/11 | -6.402 | 150.392 | Unassociated / Free school | 10 | 11/03/12 | 321 | Anchored FAD |
| A0616 | Yellowfin | 64 | Lotek - L28 | 29/01/12 | -6.166 | 150.485 | Unassociated / Free school | 10 | 26/03/12 | 322 | Unknown |
| 854 | Yellowfin | 74 | Lotek - L28 | 18/04/13 | -6.038 | 151.181 | Unassociated / Free school | 10 | 09/03/14 | 325 | Unknown |
| A0614 | Yellowfin | 64 | Lotek - L28 | 29/01/12 | -6.166 | 150.485 | Unassociated / Free school | 10 | 24/12/12 | 330 | Unassociated / Free school |
| A0613 | Yellowfin | 64 | Lotek - L28 | 29/01/12 | -6.166 | 150.485 | Unassociated / Free school | 10 | 22/02/13 | 390 | Unknown |
| A13514 | Yellowfin | 61 | Lotek - L24 | 27/02/07 | -6.314 | 150.284 | Unassociated / Free school | 300 | 23/06/07 | 455 | Unknown |
| B2645 | Yellowfin | 74 | Lotek - L23 | 27/03/07 | -5.133 | 149.171 | Anchored FAD | 60 | 25/05/07 | 462 | Unknown |

*Data Preparation*

Compression of raw tag data consisted of sectioning the time-series into bins, and calculating summary metrics from each section. As tropical tuna are known to exhibit characteristic behaviours tightly linked to both night- and day-time respectively (Matsumoto, Kitagawa, and Kimura 2013a), the first of these divisions are made at estimated dawn and dusk points. Smaller time-bins are then sectioned by dividing the time-series further between these initial crepuscular divisions. In Scutt Phillips et al. (2015), this sectioning was initially undertaken by estimating crepuscular periods based re-occurring changes in depth at two points in the day using a split-moving window analysis. The two most consistent times-of-day at which strong shifts in vertical behaviour were found, and then assumed to represent an accurate proxy for dawn and dusk over each 24-hour period of the time-series.

However, this approach ignores changes to the time of dawn and dusk that may occur during the time-at-liberty of the fish. Here, we use similar marked changes in vertical behaviour throughout a 24-hour day to form the basis of a model for each time-series that estimates how the occurrence of dawn and dusk change over time. This estimation of crepuscular timing is independent of light-at-depth data.

Two processes cause a drift in sunrise and sunset times. First, dependant on latitude, seasonal changes cause day length to increase and decrease throughout the course of the year. Second, horizontal migration by the fish causes a change in both day length (latitudinally) and time of dawn and dusk (longitudinally). **To incorporate these two processes, the timing of section bin divisions is represented by a simple linear model, which includes terms for start of the day (dawn) and length of the day-time period. Parameters are included to allow both these values to drift over time, providing a mechanism to incorporate migration and season. These drift parameters operate at a weekly timescale and are limited to additions of** ± 0.5 hours, i.e. both the start of dawn and day-time length cannot change by more than half an hour from one week to the next.

The time of the day at which dawn occurs during a given day for week ***w***, ***D_w_***, is

$$D_{w}=D+\sum_{t=1:w} {\Delta D}_{t}$$

**where *D* is the time of dawn on the first day of the time-series, and *ΔD_t_*** is the drift in occurrence of dawn associated with week ***t***.

The day-time length for a given day during week ***w***, ***L_w_***, is

$$L_{w}=L+\sum_{t=1:w} {\Delta L}_{t}$$

**where *L* is the length of the day-time on the first day of the time-series, and *ΔL****_t_* is the drift of day length associated with week ***t***. Each day of data during week **w** is binned into two “day” and “night” sections, divided by ***D_w_*** *and* ***D_w_+ L_w_***, representing estimated dawn and dusk periods. Further divisions are made equally between these boundaries to create the desired number of binned sections during a 24-hour period. These parameters were estimated by minimising the sum of the variance during the estimated day and night periods, converging on solutions that divide the time-series into two sections per 24-hour period that exhibit consistent depth profiles.

Time-series were then compressed to sequences of summary metrics over our chosen temporal scale: approximately three-hour time-bins. Given that initial day and night section binning are not necessarily equal due to the estimated times of dawn and dusk, the size of each time-bin may not be exactly three-hours. As in Scutt Phillips et al. (2015), the compressed time-series were arranged as bivariate measures of variation in depth and central tendency of water temperature experienced during each time bin, forming the observation model of behaviour in our study. Variation in depth was represented by the standard deviation of depth during each time-bin, while central tendency of water temperature was captured using the mean water temperature. These two variables capture variation in different patterns of vertical movement. Being a representation of depth (in terms of thermal habitat), and association at the depth, this description is also related to the exposure of fish to surface fishing gears. This multivariate arrangement of compressed behavioural data formed the time-series used to estimate HMM parameters.

*Surface-association Probability Threshold*

To examine the effect of varying the assumed threshold value on the number and occurrence of classified surface-associations, the proportion of each time-series defined as surface-associative was calculated, where a threshold of 0.99 was associated with very few defined events and 0.01 was where almost the entire time-series was classified as surface-associative at steps of 0.02. The effect of assuming different threshold probabilities when defining a single surface-association event resulted in the proportion of the time-series classified as this behaviour reaching an asymptote sharply after the threshold of 0.5, which represented behaviour of a typical two-state switching nature (Figure S1). For most individuals, the entire time-series was classified as surface-associated behaviour at a threshold of 0.1. The rate of change between successive threshold probabilities also appeared to include a stepwise increase, most clearly in the time series’ from bigeye tuna. This was a result of the 24-hour rolling mean window consisting of 8 time-steps, each representing 3 hours of raw data (100/8, i.e. a more likely marked change every 12.5% change in the assumed threshold probability). A marked increase in the proportion of classified surface-associations was observed at around a 0.75 threshold in the time series from bigeye tuna while time series from yellowfin tuna demonstrated a more gradual increase in gradient. This value was therefore chosen as a consistent threshold value to quantify the occurrence, length and number of individual surface-association events for all fish, representing 75% of a 24-hour period as shallow-state behaviour.

Figure A

*Example Geolocation Estimates*

As described in the main text, light-based geolocation estimates for a subset of yellowfin were combined into a single raster map at a 0.1 degree grid cell resolution. Geolocation estimates formed elliptical polygons representing the two-dimensional 95% confidence interval of an individual’s location at three points each day. Figure S2 shows a set of geolocation polygons from an example time-series, alongside a diagram of how geolocation estimates from multiple fish were combined into a raster map.


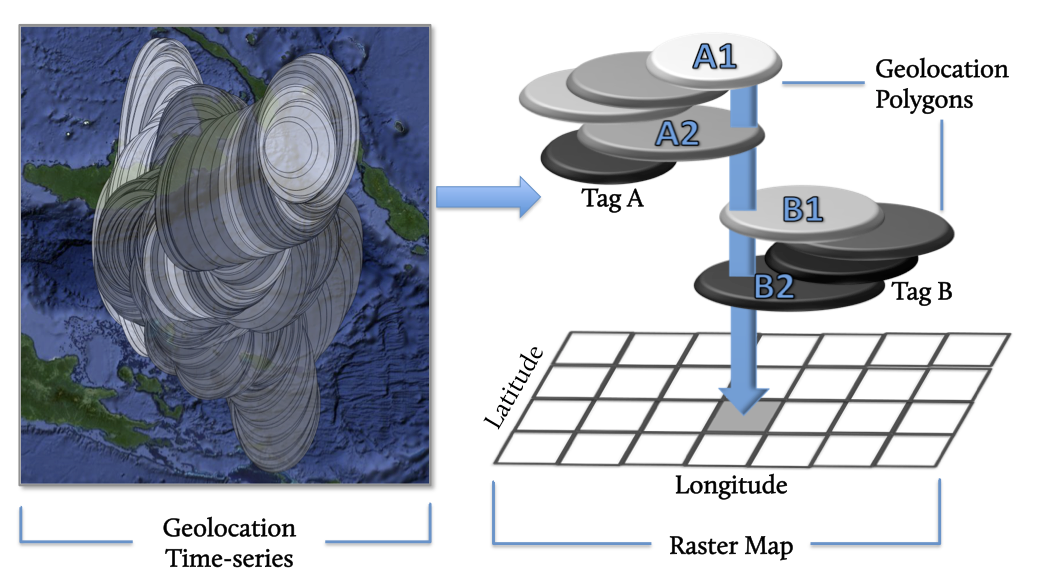


Figure B

*References*

Matsumoto T, Kitagawa T, Kimura S (2013) Vertical behavior of bigeye tuna (Thunnus obesus) in the northwestern Pacific Ocean based on archival tag data. *Fish Oceanogr* 22(3):234–246.

Scutt Phillips J, Patterson TA, Leroy B, Pilling GM, Nicol SJ (2015) Objective classification of latent behavioral states in bio-logging data using multivariate-normal hidden Markov models. *Ecol Appl* 25(5):1244–1258.
